# Inhibition of the cAMP/PKA/CREB Pathway Contributes to the Analgesic Effects of Electroacupuncture in the Anterior Cingulate Cortex in a Rat Pain Memory Model

**DOI:** 10.1155/2016/5320641

**Published:** 2016-12-20

**Authors:** Xiao-Mei Shao, Jing Sun, Yong-Liang Jiang, Bo-Yi Liu, Zui Shen, Fang Fang, Jun-Ying Du, Yuan-Yuan Wu, Jia-Ling Wang, Jian-Qiao Fang

**Affiliations:** ^1^Department of Neurobiology and Acupuncture Research, The Third Clinical Medical College, Zhejiang Chinese Medical University, Hangzhou, China; ^2^Department of Acupuncture and Moxibustion, Wenzhou Hospital Affiliated to Zhejiang University of Traditional Chinese Medicine, Wenzhou, China

## Abstract

Pain memory is considered as endopathic factor underlying stubborn chronic pain. Our previous study demonstrated that electroacupuncture (EA) can alleviate retrieval of pain memory. This study was designed to observe the different effects between EA and indomethacin (a kind of nonsteroid anti-inflammatory drugs, NSAIDs) in a rat pain memory model. To explore the critical role of protein kinase A (PKA) in pain memory, a PKA inhibitor was microinjected into anterior cingulate cortex (ACC) in model rats. We further investigated the roles of the cyclic adenosine monophosphate (cAMP), PKA, cAMP response element-binding protein (CREB), and cAMP/PKA/CREB pathway in pain memory to explore the potential molecular mechanism. The results showed that EA alleviates the retrieval of pain memory while indomethacin failed. Intra-ACC microinjection of a PKA inhibitor blocked the occurrence of pain memory. EA reduced the activation of cAMP, PKA, and CREB and the coexpression levels of cAMP/PKA and PKA/CREB in the ACC of pain memory model rats, but indomethacin failed. The present findings identified a critical role of PKA in ACC in retrieval of pain memory. We propose that the proper mechanism of EA on pain memory is possibly due to the partial inhibition of cAMP/PKA/CREB signaling pathway by EA.

## 1. Introduction

Pain memory is one of the pivotal pathogeneses of chronic pain, which is involved in sensory-discriminative, emotional affective, and cognitive evaluative pain [1, 2]. It is a nociceptive pain characterized by hyperalgesia and allodynia, resulting in formation of memories and negative emotions of pain in the brain [3–6]. This process consists of acquisition, consolidation, and retrieval of pain [7, 8]. Nociceptive sensory neurons acquire and transfer signals up to the related nuclei in the brain, including the anterior cingulate cortex (ACC), the prefrontal cortex, the hippocampus, the amygdala, and the insular cortex [1, 9–11], to form long-term memory with repeated and persistent stimulation from the emotional environment of short-term memory. Researchers have suggested that the activation of cyclic adenosine monophosphate (cAMP), protein kinase A (PKA), cAMP response element-binding protein (CREB), and their associated signaling pathways can regulate long-term synaptic plasticity to modulate both memory storage and retrieval [12, 13]. However, a clear understanding of the pain memory pathway in the ACC is still lacking.

The cAMP/PKA/CREB signaling pathway has been demonstrated to be crucial in memory formation and pain modulation [13–15]. Neuronal synaptic plasticity at the molecular, neuroanatomical and functional levels has been verified throughout the neuroaxis in response to persistent pain [1]. The activation of the cAMP/PKA/CREB signaling pathway can improve the recognition function [16] and exert an antidepressive action [17] through the enhancement of structural synaptic plasticity in the hippocampus [15, 18]. The ACC is an area that encodes pain averseness, thus contributing to pain modulation [19, 20]. As we discovered in a previous study, the phosphorylation of CREB (p-CREB) results in a profound increase in pain memory in the ACC [21]. Thus, we assume that the pathway in pain memory induces a gradual activation of cAMP, PKA, and CREB after nociceptive stimulation in the ACC and that longer lasting forms of latent long-term central sensitization promote long-term memory formation through the cAMP/PKA/CREB signaling pathway.

Due to the growing importance of pain memory in chronic pain study, it is necessary to identify measures to alleviate pain memory. Indomethacin is often used to treat inflammatory pain. However, its usage is restricted due to its side-effects and poor efficacy. Till now, there are few studies about indomethacin on pain memory. Electroacupuncture (EA), a type of acupuncture with electronic stimulation, is widely applied as analgesic for chronic pain in clinical settings. Our previous work has indicated that EA treatment can alleviate the retrieval of pain memory [21]. Although some of the pain modulation mechanisms of the analgesic effects of EA and indomethacin have been demonstrated, their potential mechanisms underlying pain memory remain unclear.

In this study, we established an animal pain memory model using two injections of carrageenan [21, 22]. Animals were treated with EA and indomethacin to study the different effects and explore the mechanism of EA on pain memory. Our data confirmed the advantageous effect of EA and further proposed that the effect of EA is partially through the inhibition of the cAMP/PKA/CREB signaling pathway.

## 2. Materials and Methods

### 2.1. Subject

Male adult Sprague-Dawley rats (Sino-British SIPPR/BK Lab. Animal Ltd., Shanghai, China) weighing 180–200 g (6 weeks) were kept at a controlled room temperature (22°C) with a 12-h light-dark cycle and free access to rodent chow and water. All animal experiments were performed according to the National Institutes of Health Guide for the Care and Use of Laboratory Animals [21, 23].

### 2.2. Pain Memory Model

As described previously [21], the pain memory model induced by two injections of carrageenan was selected to complete the study. The first carrageenan injection was placed into the left hind paw plantar surface via the subcutaneous injection of 0.1 mL of 2% carrageenan (Sigma Chemical Co, St. Louis, MO, USA) to induce acute inflammatory pain. After a 14-day recovery period, the pain value of the left hind paw was recovered. Then the second carrageenan injection was placed into the right hind paw to induce another pain. At this time, the left hind paw, which did not receive the second injection of carrageenan, appeared to exhibit nociceptive hyperalgesia. In the present study, the hyperalgesia of the left hind paw was regarded as the retrieval of pain memory [21, 22].

### 2.3. Experimental Design

Three experiments were designed and administered to study the effects of EA and indomethacin on pain memory and explore the potential mechanisms with the cAMP/PKA/CREB signaling pathway.

**Figure 1:**
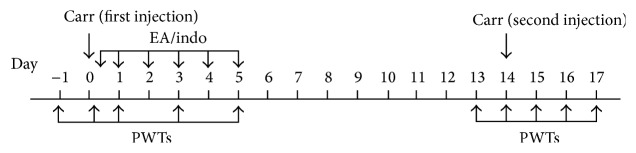
Flow chart of the procession of a pain memory model and intervening measures. The pain memory model was induced by two injections of carrageenan. The first inflammatory reaction was induced with carrageenan injection in the left hind paw at day 0. The paw withdrawal thresholds (PWTs) at days −1, 0, 1, 3, and 5 were recorded and that the one at day −1 was regarded as the baseline value. The EA/indo interventions were administered at 5 h, 1–5 d after the first carrageenan injection. The second inflammatory reaction was induced by injection in the right hind paw at day 14. The PWTs at day 13 through 17 were recorded and the one at day 13 was regarded as the new baseline value. The PWTs of the left (uninjected in the second injection of carrageenan) hind paw on day 15 through 17 manifested the memory of pain.

In Experiment 1, all rats were divided randomly into 5 groups (*n* = 10/group): the control, model, indo (indomethacin), EA, and the sham EA groups. Saline (0.9%) or carrageenan (2%) was injected into the hind paws twice at a 14 d interval. The rats in the indo, EA, and sham EA groups were treated with indomethacin, EA, and sham EA, respectively (Figure 1). The aim of this experiment was to observe the different effects of the therapies on pain memory by detecting the pain behavioral response.

**Figure 2:**
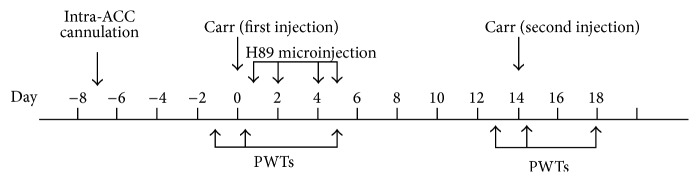
Flow chart of the PKA inhibitor intervention and PWTs detection in different time points. Before the establishment of pain memory model, intra-ACC cannulation was implanted. H89 microinjection was administered at 5 h and 1–5 d continually. The PWTs at days −1, 0, and 5 in the first injection time and days 13, 14, and 18 in the second injection time were recorded to evaluate pain.

In Experiment 2 (Figure 2), the sections of right ACC in rats were implanted with stainless steel guided cannulae and divided into 2 groups (*n* = 5/group): the model + vehicle group (implanted rats treated with the vehicle) and the model + H89 (implanted rats treated with PKA inhibitor H89). This experiment was performed to identify the role of PKA in the retrieval of pain memory and to determine whether the EA had a similar effect with H89 in the retrieval of pain memory partially.

In Experiment 3, to explore the potential mechanisms of the different effects of EA, indomethacin, and sham EA on pain memory, the right ACC tissues of the rats from Experiment 1 were prepared. The grouping was the same as Experiment 1. Four of 10 rats in each group were further used for double-immunofluorescence labeling and six others were used for western blot assays.

### 2.4. Electroacupuncture

Without anesthesia, EA treatment was applied in rats of the EA group. In a previous study [21], we found that EA treatment at bilateral acupoints “Zusanli” (ST36) and the reference electrode (1 cm inferior of “Zusanli”) were effective for impairing the retrieval of a pain memory. Therefore, we used the same methods including the acupoints and EA parameters in this study. The acupoints were needled with stainless acupuncture needles (0.25 mm in diameter × 13 mm in length) and electrically stimulated with a HANS Acupuncture Point Nerve Stimulator (LH-202H; Huawei Co., Ltd., Beijing, China). The parameters of EA were set as follows: 2/100 Hz of frequency with automatically shifting between 2 Hz and 100 Hz stimulation for 3 s each; a square wave current output (pulse width: 0.2 ms); an intensity range of about 1-2 mA adjusted to animals' local muscle contractions. The treatment was administered at 5 h, 1–5 d after the first carrageenan injection. To confirm the authenticity of the effects of EA, thin stainless acupuncture needles (0.18 mm in diameter × 13 mm in length) pierced the subcutaneous acupoints of the sham EA animals without electrical stimulation. The animals wore black hoods over their heads for a calming effect during the administration of EA. No signs of stress were observed. To maintain consistency, the rats in the model and control groups also wore black hoods at the same time.

### 2.5. Behavioral Pain Test

Mechanical allodynia, assessed by the paw withdrawal thresholds (PWTs), was used to evaluate the pain response. An UGO-Basile Dynamic Plantar Aesthesiometer (UGO 37450, Milan, Italy), with an improved calibrated von Frey filaments method [21, 24, 25], was operated by a trained investigator who was blind to the experimental allocation. One measurement was taken at 5 min intervals, for a total 5 measurements, and the last 4 were averaged as the PWTs value. All behavioral pain tests were performed between 9:00 and 15:00. All rats underwent habituation to the environment and testing procedure 2 days before the formal experiment.

### 2.6. Drug Administration

H89 (Sigma), a PKA inhibitor, was dissolved in dimethyl sulfoxide (DMSO) and water (10 *μ*M, the ratio of DMSO and water is 1 : 999). Rats in the model + H89 group in Experiment 2 received H89 at a concentration of 10 *μ*M via intra-ACC cannulation and brain microinjection. The specific times were the same as the times of EA administration in Experiment 1. Indomethacin (Yun Peng Co., Ltd., Shanxi, China) was dissolved in 0.9% saline and ingested by intragastric administration (3 mg/kg) for 6 times. The specific ingestion times were the same as the EA administration times.

### 2.7. Intra-ACC Cannulation and Microinjection

For PKA inhibitor infusion, intra-ACC cannulation and microinjection were performed as previously reported [20, 26]. Briefly, rats in Experiment 2 were anesthetized with 7% chloral hydrate (500 mg/kg, intraperitoneally) and fixed in a stereotaxic apparatus (RWD, Life Science Co., Ltd, Shenzhen, China). After the exposure of the skull around the bregma, the stainless steel guide cannula (3 mm long) was implanted according to the following coordinates in the right ACC: 3.2 mm anterior to the bregma, 0.6 mm on the right side of the midline, and 1.5 mm below the top of the skull [11, 21]. Then, the cannula was fixed to the skull with 3 screws and dental acrylic. A stylet 0.5 mm longer than the cannula was inserted to keep it in place prior to the microinjection. All rats were allowed at least 7 days to recover.

The intra-ACC microinjections were administered through the implanted guide cannulae using injection needles (3.5 mm long) that were connected by a polyethylene tube fitted to a 100 *μ*L Hamilton microsyringe. The volume of the drug solution to be injected into the ACC of the rats in the model + H89 group was 1 *μ*L and the duration of the drug infusion was 1 min. As a control, the vehicle containing DMSO was injected into the ACC of the rats in the model + vehicle group.

### 2.8. Tissue Preparation

At day 18, after the behavioral pain test, the rats were anesthetized with 10% chloral hydrate (350 mg/kg, intraperitoneally) and perfused via cardiac puncture with 0.9% saline. The right ACC tissues of the rats were removed immediately for the western blot assays. Subsequently, after perfusion with 4% paraformaldehyde, the whole brains of the other rats were harvested and post fixed by immersion in the same fixative for 24 h and dehydrated in a 15%–30% sucrose gradient for 24 h for immunofluorescence.

### 2.9. Double-Immunofluorescence Labeling

Immunofluorescence was used to evaluate the levels of cAMP, phosphorylation of PKA (p-PKA), and p-CREB expression in the right ACC. Furthermore, we used double-immunofluorescence labeling to demonstrate correlations among the above proteins as previous description [21]. Coronal brain slices 30 *μ*m in thickness taken from the bregma at 3.2 mm containing the ACC were washed with 0.01 M PB saline (PBS, pH 7.4) and blocked with 5% goat serum. The tissue sections were then incubated overnight with a rabbit anti-PKA (phospho T197) antibody (p-PKA, 1 : 200; Abcam, Boston, MA, USA) and either a mouse anti-cAMP antibody (1 : 1000; Abcam) or a mouse anti-phospho-CREB (Ser133) polyclonal antibody (p-CREB, 1 : 200; Millipore, Billerica, MA, USA). After rinsing in PBS, the sections were incubated in a goat-anti-mouse Alexa Fluor 488-conjugated secondary antibody (1 : 200; Jackson Immuno Labs, West Grove, PA, USA) and a goat anti-rabbit Alexa Fluor 594-conjugated secondary antibody (1 : 200; Abcam). Fluorescence signals were detected by a Nikon A1R laser scanning confocal microscope. The positive cells were counted and analyzed by NIS elements AR software.

### 2.10. Western Blot

Western blot was used to detect the protein expression levels of cAMP, p-PKA, and p-CREB in the right ACC. The tissues were weighed and homogenized in RIPA buffer (Beyotime P0013B, Haimen, Jiangsu, China) plus protease inhibitors. After centrifugation, a BCA protein assay kit (Beyotime P0012S, Haimen, Jiangsu, China) was used to determine the protein concentrations. Protein samples (20 *μ*g) from each group were separated by 10% SDS-PAGE and transferred onto polyvinylidene fluoride membranes (Bio-Rad, Hercules, CA, USA). The membranes were blocked in 5% nonfat milk followed by incubation with primary antibodies: rabbit anti-p-PKA (1 : 5000; Abcam), rabbit anti-p-CREB (1 : 1000; Millipore), and rabbit anti-*β*-actin (1 : 5000; Abcam). Then, the membranes were incubated with secondary anti-rabbit HRP-conjugated IgG antibody (1 : 20000, Abcam). The immunoreactive bands were visualized by using an Immun-Star™ HRP Chemiluminescence Kit (Bio-Rad). The relative intensity of each band for *β*-actin was measured by an ImageQuant LAS 4000 system (GE Healthcare, Hino, Japan) and was analyzed by ImageQuant TL software (version 7.0, GE Healthcare).

### 2.11. Statistical Analysis

The data are expressed as the means ±SEM. Comparisons among different groups were performed by one-way, repeated-measures analysis of variance (ANOVA). For post hoc analysis, the least significant difference (LSD) and Dunnett's test were used for equal or unequal variances, respectively, as examined by the homogeneity of the variance test. Comparisons between two groups were evaluated using independent-sample *t* tests or paired-sample *t* tests. All statistical analyses were considered significant at the level of *p* < 0.05.

## 3. Results

### 3.1. Electroacupuncture Alleviated the Retrieval of Pain Memory in Model Rats

**Figure 3:**
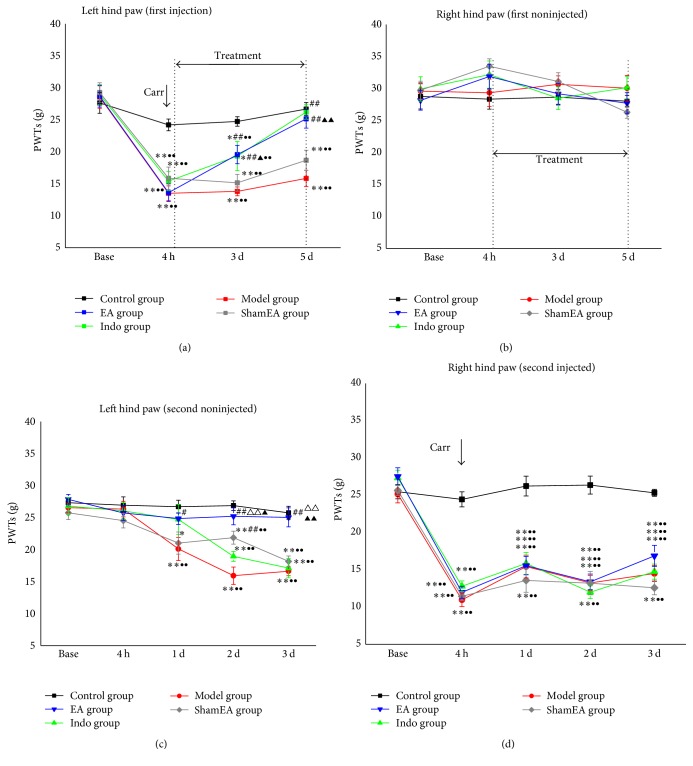
EA alleviated the retrieval of pain memory. The rats in the pain memory model were divided into the control, model, indo, EA, and sham EA groups, and the mechanical allodynia in each group was examined (*n* = 10). (a) On the left hind paw (first injected), EA or indomethacin treatment increased the PWTs significantly compared with the model group. (b) There was no significant difference in all groups at the same period. (c) On the left hind paw (second noninjected), the PWTs in the model group decreased significantly compared with the control group and its baseline. EA treatment administered at the first injection time point inhibited this progress. (d) The second injection of carrageenan into the right hind paw significantly decreased the PWTs of the carrageenan-injected groups compared with the control group and their baselines. Error bars indicate the SEM. ^**∗**^ *p* < 0.05 and ^**∗∗**^ *p* < 0.01, versus the control group; ^#^ *p* < 0.05 and ^##^ *p* < 0.01, versus the model group; ^△△^ *p* < 0.01, versus the indo group; ^▲^ *p* < 0.05 and ^▲▲^ *p* < 0.01, versus the sham EA group; ^●●^ *p* < 0.01, versus baseline.

The pain memory model that we used in this experiment was described by Kissin et al. [22] and had been used successfully in previous experiments [21]. The PWTs were detected after two carrageenan injections separated by a 14 d interval in Experiment 1. Firstly, we measured the PWTs at the first injection time point to observe the effects of EA on acute inflammatory pain. As expected, the baseline values of all of the groups were similar. At 4 h after the carrageenan injection, the PWTs of the left hind paw in all groups except the control were significantly decreased compared with the control group (*p* < 0.01). After treatments, at 5 h and 1–5 d after the first carrageenan injection continuously, the PWTs of the left hind paw in the EA and indo groups increased significantly at 3 d and 5 d compared with the model group (*p* < 0.01). There were no significant differences between the two groups. The PWTs of the EA group also significantly increased compared with the sham EA group at 5-d time point (*p* < 0.01). Compared with the baseline values, the PWTs of each time point in the model, indo, EA, and sham EA groups significantly decreased (*p* < 0.01), except in the EA and indo group at the 5-d time point (Figure 3(a)). The noninjected (right) hind paw showed normal and stable levels of PWTs values with treatment at the same time points (Figure 3(b)).

Next, we investigated the analgesic effects of the preadministration of EA on pain memory by recording the PWTs of the left hind paws at the second injection time point. Note that the PWTs of all groups returned to their baseline values after a 14 d interval.  Figure 3(c) presents the different changes in the PWTs of the noninjected (left) hind paws from the 5 groups at 4 h, 1 d, 2 d, and 3 d after the second carrageenan injection into the right hind paw. The values of the control group remained normal, whereas the model group exhibited a profound decrease from the 1 d to the 3 d time point compared with the control group (*p* < 0.01). This was regarded as the retrieval of pain memory. However, the PWTs in the EA group did not decrease and maintained similar levels with those of the control group. The values from the indo and sham EA groups also decreased. When compared with the model, indo, and sham EA groups, the PWTs of the EA group significantly increased at 3 d time point (*p* < 0.01). Compared with the baseline values of the model group, decreased PWTs were observed at 1 d to 3 d (*p* < 0.01). Similar decreased values appeared at 2 d and 3 d in the indo and sham EA groups compared with their baseline values (*p* < 0.01). However, there was no significant difference among values at different time points in the EA group. Because there was no intervention at the second injection time point, the injected (right) hind paws of the model, indo, EA, and sham EA groups showed significantly decreased PWTs values at 4 h after injection until 3 d compared with the control group and their separated baseline values (*p* < 0.01). The preadministration of EA had no effect on PWTs compared with the model group (Figure 3(d)).

### 3.2. Intra-ACC Microinjection of a PKA Inhibitor Blocked the Occurrence of Pain Memory

**Figure 4:**
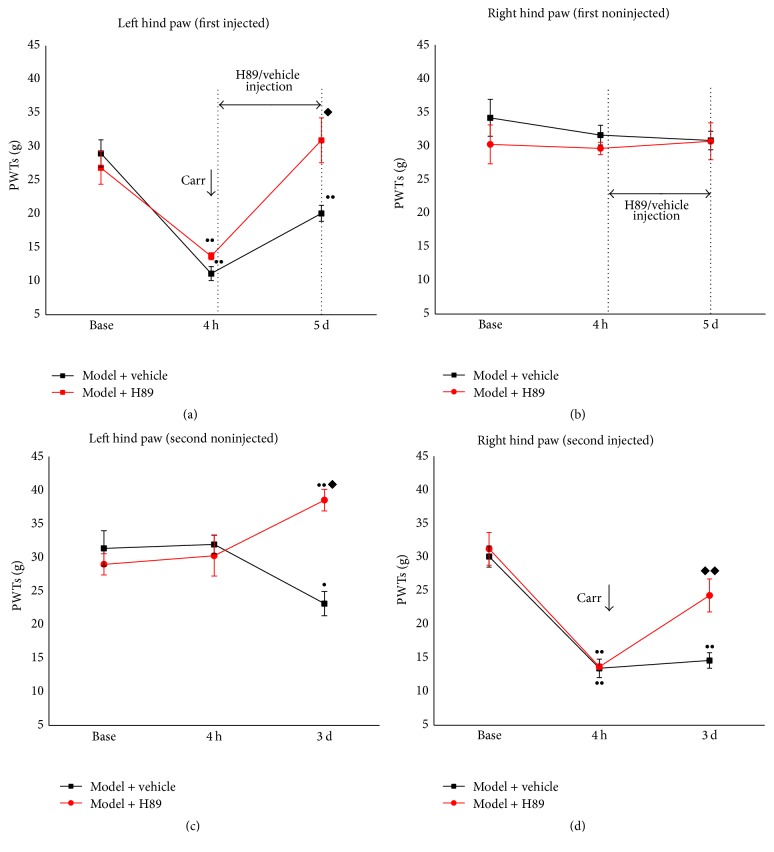
The effects of the PKA inhibitor of pain memory model. The rats were divided into the model + vehicle and the model + H89 groups and the mechanical allodynia was examined (*n* = 7). (a) In the left hind paw (first injected), the PKA inhibitor intervention significantly increased the PWTs from acute inflammatory pain. (b) In the right hind paw (first noninjected), the PWTs of the two groups were not significantly different. (c) In the left hind paw (second noninjected), PKA inhibitor intervention significantly increased the PWTs from pain memory. (b) In the right hind paw (second injected), PKA inhibitor intervention significantly increased the PWTs compared with the model + vehicle group. Error bars indicated SEM. ^*◆*^ *p* < 0.05 and ^*◆◆*^ *p* < 0.01, versus the model + vehicle group; ^●^ *p* < 0.05 and ^●●^ *p* < 0.01, versus baseline.

To elucidate the key role of PKA in the retrieval of pain memory, as well as to explore the potential mechanism of EA treatment, a PKA inhibitor (H89) was delivered into the ACC by intra-ACC cannulation and microinjection. The pain memory model with a vehicle group was set up as the control group to compare with the H89 group. The procedures were similar to those in Experiment 1: the pain memory model was established in the two groups of rats and was followed by a PKA inhibitor intervention at the same time points with EA treatment. As shown in Figure 4(c), the vehicle-injected rats presented with pain memory via decreased PWTs in the left hind paw 3 d after the second carrageenan injection was administered into the right hind paw compared with baseline (*p* < 0.05). H89 inhibited the retrieval of pain memory significantly, with increased PWTs compared with the model + vehicle group and its baseline values (*p* < 0.01). In Figure 4(d), the PWTs of the right hind paw (the second injection) of the H89-treated rats showed an increase at 3 d after the carrageenan injection (*p* < 0.01).

### 3.3. Electroacupuncture Reduced the Activation of cAMP, PKA, CREB, in the ACC in Model Rats

**Figure 5:**
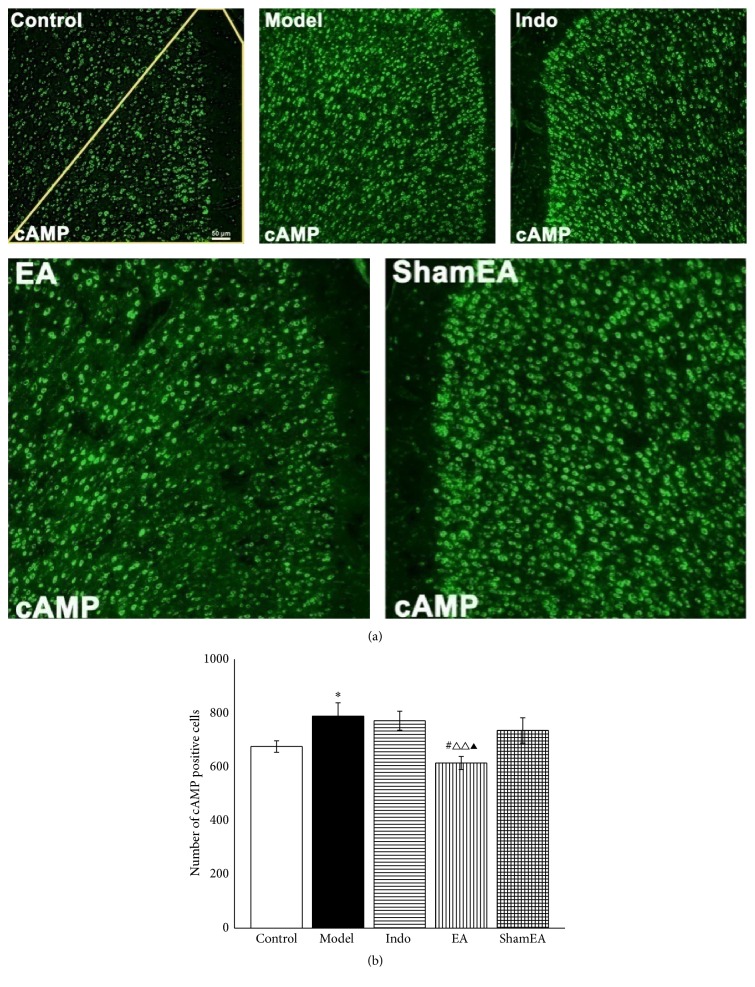
EA inhibited the activation of cAMP in the ACC of pain memory model rats. The right ACC tissues from the control, model, indo, EA, and sham EA groups were prepared and labeled with immunofluorescence (*n* = 3, five slides for each rat). (a) Views of cAMP-positive cells (scale bar, 50 *μ*m). The yellow line indicates the field in which the positive cells were counted. (b) Quantification of cAMP-positive cells showed that EA treatment at the first injection time point significantly reduced the activation of cAMP. Error bars indicated SEM. ^**∗**^ *p* < 0.05, versus the control group; ^#^ *p* < 0.05, versus the model group; ^△△^ *p* < 0.01, versus the indo group; ^▲^ *p* < 0.05, versus the sham EA group.

It has been shown that the cAMP/PKA/CREB signaling pathway is crucial to pain [14, 27] and memory formation in the ACC [26], which may occur via brain neural synaptic plasticity. To explore the different effects of EA and indomethacin on the cAMP/PKA/CREB signaling pathway in pain memory, we first examined the changes in cAMP, p-PKA, and p-CREB protein expression separately in the ACC via immunofluorescence and western blot assays. The numbers of cAMP-positive cells in the model group increased significantly compared with those of the control group (*p* < 0.05). After treatment with EA, indomethacin, or sham EA, the EA treatment profoundly decreased the number of positive cells compared with those in the model (*p* < 0.05), indo (*p* < 0.01), and sham EA (*p* < 0.05) groups. However, indomethacin and the sham EA treatment did not show this effect (Figures 5(a)-5(b)).

**Figure 6:**
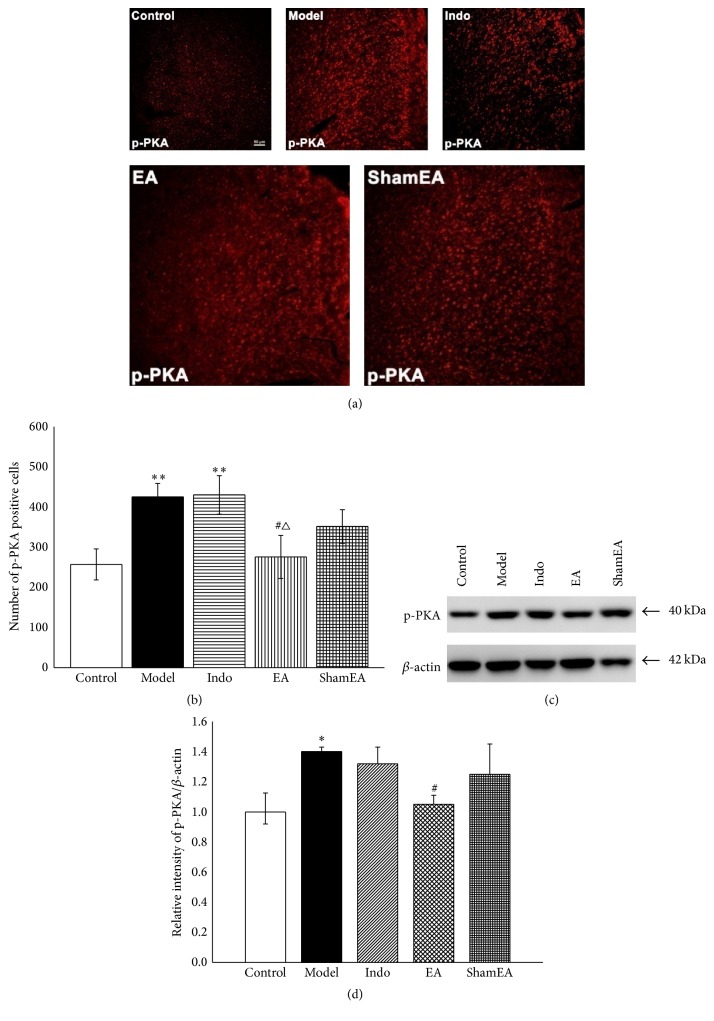
EA decreased the phosphorylation of PKA in the ACC of pain memory model rats. The right ACC tissues of different groups were prepared and labeled with immunofluorescence (*n* = 3, five slides for each rat). Total protein extracts from the ACC tissues were detected by western blotting (*n* = 6). (a) Views of p-PKA-positive cells in the ACC (scale bar, 50 *μ*m). (b) Quantification of p-PKA-positive cells showed that EA treatment at the first injection time point significantly reduced the amount of positive p-PKA. (c) Western blotting analysis of p-PKA in the different groups. (d) Quantitative analysis using the western blot assay showed that EA significantly reduced the phosphorylation levels of PKA. Error bars indicated SEM. ^**∗**^ *p* < 0.05 and ^**∗∗**^ *p* < 0.01, versus the control group; ^#^ *p* < 0.05, versus the model group; ^△^ *p* < 0.05, versus the indo group.

Because resting PKA and CREB contribute minimally to the synaptic plasticity of the learning and memory processes, the phosphorylation levels of PKA and CREB were examined. After the successful establishment of a pain memory, the amount of phosphorylated PKA (p-PKA) in the model group clearly increased compared with control group (*p* < ⁡0.01). After EA but not indomethacin or sham EA treatment, the number of positive cells in EA group decreased compared with the model (*p* < 0.05) and indo (*p* < 0.05) groups (Figures 6(a)-6(b)). The western blot results also indicated similar trends in p-PKA expression (Figure 6(c)). Quantitative analysis indicated that the relative intensity of p-PKA/*β*-actin was significantly increased in the model group compared with the control group (*p* < 0.05, Figure 6(d)). Compared with model group, EA group showed reduced relative intensity of p-PKA/*β*-actin (*p* < 0.05, Figure 6(d)).

**Figure 7:**
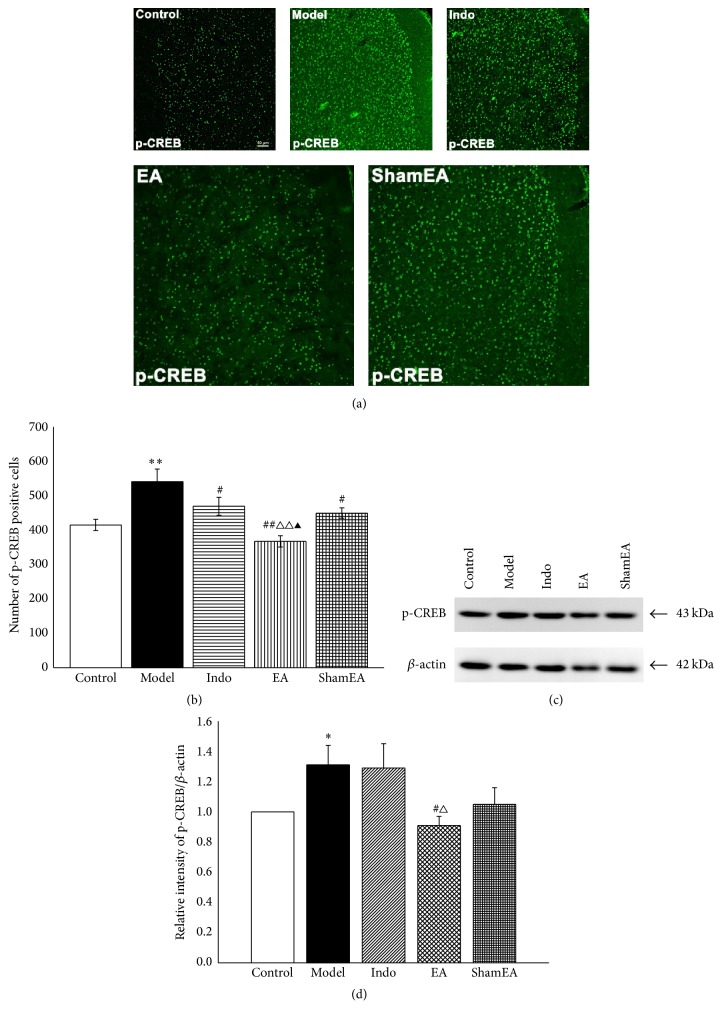
EA decreased CREB phosphorylation in the ACC of pain memory model rats. The right ACC tissues of different groups were prepared and labeled with immunofluorescence (*n* = 3, five slides for each rat). Total protein extracts from the ACC tissues were detected by western blotting (*n* = 6). (a) Views of p-CREB-positive cells in the ACC (scale bar, 50 *μ*m). (b) Quantification of p-CREB-positive cells showed that EA treatment at first injection time point significantly reduced the amount of positive p-CREB. (c) Western blotting analysis of p-CREB in the different groups. (d) Quantitative analysis using the western blot assay showed that EA significantly reduced phosphorylation level of CREB. Error bars indicated SEM. ^**∗**^ *p* < 0.05 and ^**∗∗**^ *p* < 0.01, versus the control group; ^#^ *p* < 0.05 and ^##^ *p* < 0.01, versus the model group; ^△^ *p* < 0.05 and ^△△^ *p* < 0.01, versus the indo group; ^▲^ *p* < 0.05, versus the sham EA group.

CREB is a key nuclear transcription factor for memory formation [13, 15]. Phosphorylation of CREB in the model group was increased compared with the control group (*p* < 0.01, Figures 7(a)-7(b)). The positive numbers of phosphorylated CREB (p-CREB) in EA group were decreased significantly compared with the model (*p* < 0.01), indo (*p* < 0.01), and sham EA (*p* < 0.05) groups. Indomethacin and sham EA treatment also resulted in decreased values of p-CREB (*p* < 0.05, Figures 7(a)-7(b)). The western blot quantitative analysis indicated that the relative intensity of p-CREB/*β*-actin was increased in the model group compared with the control group (*p* < 0.05) and that EA treatment significantly reduced the intensity of p-CREB/*β*-actin compared with the model (*p* < 0.05) and indo (*p* < 0.05) groups (Figures 7(c)-7(d)).

### 3.4. Colocalization of cAMP and p-PKA Was Reduced by Electroacupuncture in the ACC of Pain Memory Model Rats

**Figure 8:**
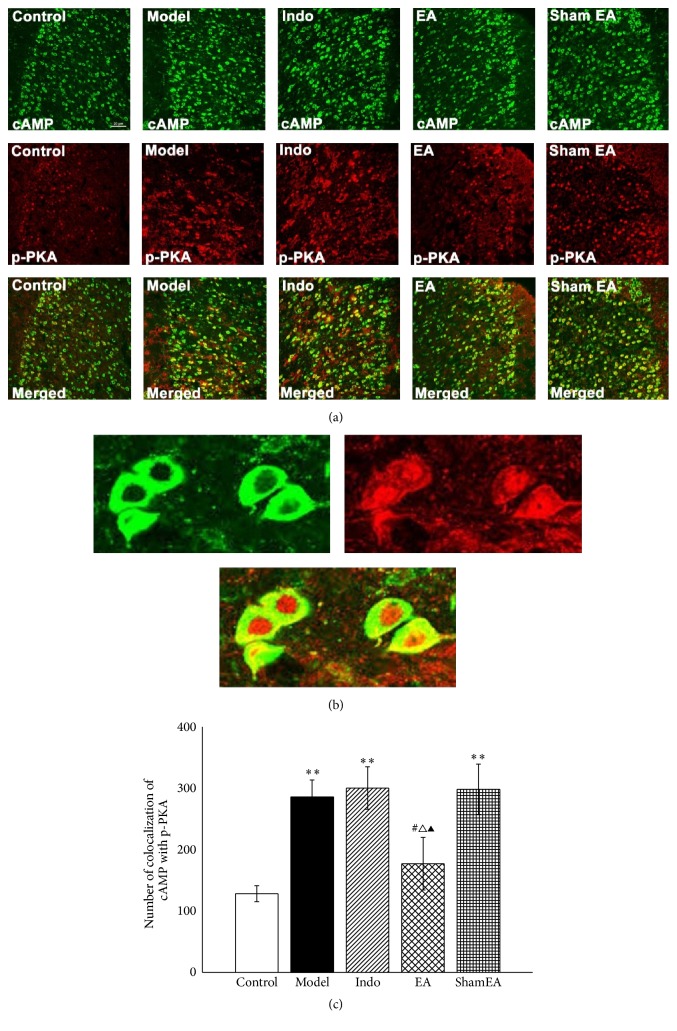
EA inhibited the colocalization of cAMP with p-PKA in the ACC of pain memory model rats. The right ACC tissues from each group were prepared and labeled with double-immunofluorescence (*n* = 3, five slides for each rat). (a) Views of the colocalization of cAMP with p-PKA (scale bar, 20 *µ*m). (b) The magnified image shows the colocalization of cAMP with p-PKA. (c) Double-labeled immunofluorescence analysis demonstrated that EA significantly reduced the amount of colocalized cAMP and p-PKA. Error bars indicated SEM. ^**∗∗**^ *p* < 0.01, versus the control group; ^#^ *p* < 0.05, versus the model group; ^△^ *p* < 0.05, versus the indo group; ^▲^ *p* < 0.05, versus the sham EA group.

Next, we detected the colocalization of cAMP and p-PKA in the ACC to elucidate whether cAMP was coexpressed with p-PKA in the pain memory model and the different effect of EA treatment on the coexpression. Double-immunofluorescence labeling for cAMP and p-PKA showed that cAMP colocalized with p-PKA in the ACC of pain memory model rats (Figure 8(a)). Because cAMP exists in the cytoplasm but p-PKA exists in the cytoplasm and nucleus, their colocalization occurs in the same plane but not at the same site (Figure 8(b)). The amount of colocalized cAMP and p-PKA increased in the model, indo, and sham EA groups compared with the control group (*p* < 0.01). A decreased amount of cAMP coexpressed with p-PKA was observed in the EA group compared with the model, indo, and sham EA groups (*p* < 0.05) (Figure 8(c)).

### 3.5. Colocalization of p-PKA and p-CREB Was Reduced by Electroacupuncture in the ACC of Pain Memory Model Rats

**Figure 9:**
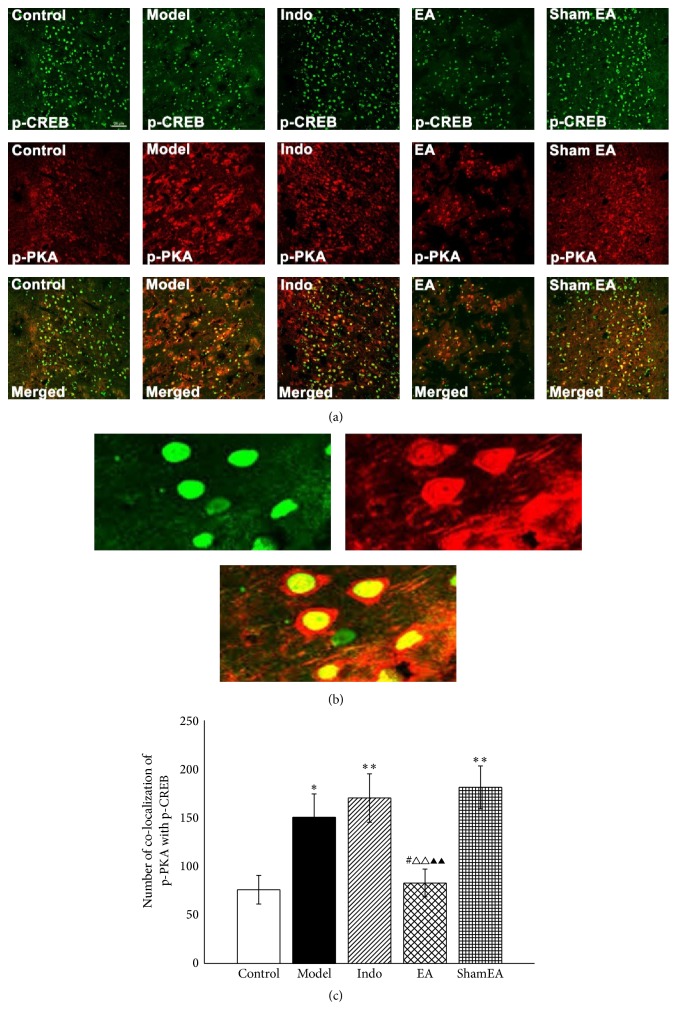
EA inhibited the colocalization of p-PKA with p-CREB in the ACC of pain memory model rats. The right ACC tissues from each groups were prepared and labeled with double-immunofluorescence (*n* = 3, five slides for each rat). (a) Views of the colocalization of p-PKA with p-CREB (scale bar, 20 *μ*m). (b) The magnified image shows the colocalization of p-PKA with p-CREB. (c) Double-labeled immunofluorescence analysis demonstrated that EA significantly reduced the numbers of colocalized p-PKA and p-CREB. Error bars indicated SEM. ^**∗**^ *p* < 0.05 and ^**∗∗**^ *p* < 0.01, versus the control group; ^#^ *p* < 0.05, versus the model group; ^△△^ *p* < 0.01, versus the indo group; ^▲▲^ *p* < 0.01, versus the sham EA group.

As described above, the colocalization of p-PKA and p-CREB in the ACC was detected to observe the effect of EA on the coexpression of p-PKA and p-CREB. Double labeling showed that p-PKA and p-CREB were coexpressed in the nuclei of the ACC (Figure 9(a)). CREB is activated in the nucleus of the ACC via cAMP/PKA signaling in pain memory.  Figure 9(b) presents the overlap of p-PKA and p-CREB colocalization on the same plane and same site. The amount of colocalization of p-PKA and p-CREB of the model (*p* < 0.05), indo (*p* < 0.01) and sham EA (*p* < 0.01) groups had a profound increase compared with the control group. EA treatment decreased the colocalization of p-PKA and p-CREB compared with the model (*p* < 0.05), indo (*p* < 0.01), and sham EA (*p* < 0.01) groups.

## 4. Discussion

The key findings of this study are that EA can alleviate the pain memory induced by two carrageenan injections, whereas indomethacin failed to do so. Furthermore, pain memory can be blocked by PKA blockage, which may indicate the relationship between pain memory and the cAMP/PKA/CREB pathway. EA can inhibit the cAMP/PKA/CREB signaling pathway partially, but NSAIDs cannot. To our knowledge, this is by far the first study that investigated the different effects and mechanisms of EA and NSAIDS on pain memory.

Pain perception is determined by 3 dimensions consisting sensory-discriminative, affective-motivational, and evaluative-cognitive modulations. Nociception activate the above 3 dimensions of pain control system for the formation of pain memory through integrated process in brain nucleus. The sensory-discriminative and affective-motivational information of nociception could induce a profound process, like the recognition, memory, attention, expectation, and so on. It should also be noted that pain sensory could be affected by pain memory vice versa [28]. Through the top-down manipulation, the information from evaluative-cognitive modulation could exert pain perception on the others [3, 29]. Actually, pain memory is a complex perception experience.

In clinic, physicians have found that pain memory is accompanied by complex regional pain syndrome [6], phantom pain [30], postoperative pain [31], and long histories of chronic pain [32]. To explore the effective therapy and the potential mechanisms of pain memory, affective-motivational and evaluative-cognitive pain have increasingly gained researchers' attentions [2, 3, 26, 28, 33]. EA, an effective pain treatment that is considered to be a mind-body therapy, has a wide range of data supporting its analgesic role and capacity to regulate mood disorder and memory [2, 23, 34, 35]. In our previous studies, we have found that the retrieval of pain memory can be alleviated by pretreatment with EA, which is partially attributed to the downregulated expression of p-CREB [21]. In the present study, our results have identified different effects of EA and NSAIDS on pain memory. The retrieval of pain memory was demonstrated by the decreased PWTs of uninjected hind paw in the second carrageenan injection. EA blocked the retrieval of pain memory by showing unchanged PWTs values, while NSAIDs treatment did not. It is suggested that the mechanism of EA on pain memory is different from NSAIDs. EA intervention not only has relieved the acute inflammatory pain, but also inhibited the retrieval of pain memory integrated in high-level central neural system. Therefore, EA has an advantage over NSAIDs in treating inflammatory pain accompanied with pain memory.

It has shown that neuronal synaptic plasticity promoted the memory and chronic pain [36, 37]. Thus, we inferred that the potential mechanism of pain memory is closely related with neuronal synaptic plasticity and long-term central sensitization. Some studies have reported that the cAMP/PKA/CREB signaling pathway critically contributes to the neuronal synaptic plasticity in the processes of pain and memory [15, 27, 38]. Being a crucial second messenger, cAMP is involved in varies types of learning and memory processes and long-term synaptic plasticity [17, 39]. Years ago, it was found that increased levels of cAMP in sensory neurons indeed lead to a transient enhancement of transmitter release in the synaptic connection between sensory and motor neurons, which was regarded as a biochemical mechanism for short-term and long-term changes [13, 40]. Some studies have suggested that signaling pathways involving cAMP participate in inflammation and injury sensitization [41, 42]. Here, our study found an increased number of cAMP-positive cells in the ACC of pain memory model rats. Furthermore, when compared with indomethacin and sham EA intervention, EA treatment showed a profound advantage in reducing the number of cAMP-positive cells. In all, these results showed that the role of cAMP as a relative upstream protein in pain memory can be inhibited by EA treatment.

Thus, it would be interesting to determine whether the decreased release of cAMP by EA treatment can affect downstream processes. Reports from earlier studies have demonstrated that cAMP mediates almost all of its actions through PKA [13, 40]. When cAMP increases in cells, the cAMP-binding regulatory subunit of PKA is freed from the catalytic subunit of PKA and is allowed to phosphorylate its substrates [43]. A similar experiment performed in 1980 injected a catalytic subunit of PKA directly into the sensory neurons and found that PKA also sufficiently enhances transmitter release for synaptic plasticity in long-term memory [13, 44]. The inhibition of PKA activity reduces the phosphorylation of CREB [26, 45]. Other studies have indicated that different degrees of emotional/fear memories can be abolished by PKA deficiencies and that the cAMP/PKA signaling pathway is important for memory consolidation [15, 39]. The mechanical allodynia caused by inflammation and nerve injury can be inhibited by microinjections of PKA inhibitor [19, 46]. In Experiment 2 (Figure 4) of the present study, the behavioral test demonstrated that PKA inhibitor diminished the mechanical allodynia in the pain memory model. Regarding the protein expression of PKA in the ACC, the results of this study indicated that pain memory is induced by the phosphorylation of PKA, whereas EA inhibits this process. The effects of the PKA inhibitor suggested that EA treatment has a similar effect with PKA inhibitor.

cAMP and PKA serve as important proteins for the formation of memory [14, 17]. With regard to short-term memory switching to long-term memory, the synthesis of new proteins is required [13]. CREB, a cellular transcription factor that binds the cAMP response element, has a pivotal role in memory storage [9]. The activation of CREB is also closely related to pain [10, 47]. After its phosphorylation by PKA and other proteins, CREB functions as a transcriptional activator to modulate pain, long-term memory, and other physiological and pathological processes. Evidence from our previous study has shown that the phosphorylation of CREB in the ACC of pain memory model rats is significantly related to the retrieval of pain memory, and EA can influence the number of positive cells and active levels of p-CREB in the ACC [21]. In CFA-induced chronic pain rats, the levels of phosphorylated CREB are enhanced in the ACC, which suggests that the presence of synaptic plasticity is related to memories [48, 49]. The CREB-mediated response to stimuli, which acts a downstream of the cAMP/PKA pathway, can be modulated by PKA and its phosphorylation. Active levels of p-CREB in this study demonstrated that p-CREB indeed plays a key role in the retrieval of pain memory. Indomethacin and sham EA reduced the number of positive cells to some extent. However, EA treatment reduced the number of active cells and the phosphorylation levels of CREB to a much greater extent.

To verify the effects of EA treatment on pain memories were mediated through the cAMP/PKA/CREB signaling pathway; the coexpression of both cAMP and p-CREB with p-PKA in the ACC was assessed by double-immunofluorescence labeling. Our results showed that cAMP and p-PKA are coexpressed in cells but not at the same site because of their different distributions. This result indicated that the persistent increase in cAMP induces a longer enhancement of transmitter release in the synaptic connections in neurons and causes the catalytic subunit of PKA to become activated and phosphorylated [13]. Subsequently, the increased p-PKA transmits to the nucleus, where it phosphorylates the transcription factor CREB and activates the gene expression required for the formation of long-term memory [50]. The images from our study showed the coexpression of p-PKA and p-CREB at the same site in cell nuclei. Our data indicated that the amounts of coexpressed p-PKA and cAMP as well as p-CREB were increased in the pain memory model, even under conditions of indomethacin or sham EA treatments, but EA treatment could reverse this effect. In all, these results suggested that the analgesic effects of EA may be mediated via the cAMP/PKA/CREB signaling pathway, which contributes to the allodynia caused by pain memory.

## 5. Conclusions

This study is the first to demonstrate that the analgesic effect of EA in a rat pain memory model is different from that of the NSAIDs. EA has an advantage over NSAIDs in treating chronic inflammatory pain accompanied with pain memory. The present findings further identify that PKA in ACC plays a critical role in pain memory retrieval. We propose that the proper mechanism of EA on pain memory is possibly due to the partial inhibition of cAMP/PKA/CREB signaling pathway by EA. Therefore, EA may be a preferred therapy for chronic pain induced by pain memory in clinic.
